# *Laguncularia racemosa* leaves indicate the presence of potentially toxic elements in mangroves

**DOI:** 10.1038/s41598-023-31986-x

**Published:** 2023-03-24

**Authors:** Cristiane Pimentel Victório, Mayara Silva dos Santos, Aimêe Cordeiro Dias, João Pedro Silvério Pena Bento, Bruno Henrique dos Santos Ferreira, Marcelo da Costa Souza, Naomi Kato Simas, Rosani do Carmo de Oliveira Arruda

**Affiliations:** 1 Laboratório de Pesquisa em Biotecnologia Ambiental, Programa de Pós-Graduação em Ciência e Tecnologia Ambiental, Universidade do Estado do Rio de Janeiro (UERJ-ZO), Av. Manuel Caldeira de Alvarenga 1.203, Rio de Janeiro, RJ 23070-200 Brazil; 2 Laboratório de Anatomia Vegetal, Instituto de Biociências, Universidade Federal do Mato Grosso do Sul (UFMS), Campo Grande, MS 79070-900 Brazil; 3 Herbário RBR, Departamento de Botânica, Universidade Federal Rural do Rio de Janeiro (UFRRJ), Seropédica, RJ 23897-000 Brazil; 4 Laboratório de Fitoquímica, Departamento de Produtos Naturais e Alimentos, Faculdade de Farmácia, Universidade Federal do Rio de Janeiro (UFRJ), Rio de Janeiro, RJ 21941-590 Brazil

**Keywords:** Plant sciences, Environmental sciences

## Abstract

Brazilian mangroves have been severely impacted by metallurgical, petrochemical, pyrometallurgical smelters and other industrial activities. In Rio de Janeiro, mangroves are part of the Atlantic Rainforest now under the stress of high levels of industrial waste. Therefore, this work aimed to detect potentially toxic elements (PTEs) by evaluating the leaves of *Laguncularia racemosa* (L.) Gaertn. f. collected from three mangroves with different levels of pollution. To gain further insight toward an accurate diagnosis of the effects of anthropogenic pollution on mangrove stands, we evaluated leaf epicuticular wax composition, as well as morphological and anatomical traits. Samples were analyzed using inductively coupled plasma-optical emission spectroscopy (ICP-OES), gas chromatography (GC) and microscopy. Results revealed variation in the contents of PTEs among the three mangroves from lowest to highest concentration, as follows: Al (0.30–0.73), Pb (0.095–0.325) and Zn (0.25–0.30) mg/kg. Zn was detected in sclerenchyma tissues. Leaf epicuticular wax contained more than 50% of triterpenes, in particular, the pentacyclic triterpenes lupeol (41.61–55.63%) and β-amyrin (8.81–16.35%). Such high concentrations promote the increase in leaf permeability to salts and PTEs. Micromorphology of leaf epicuticular wax in *L. racemosa* also varied among the three evaluated sites, especially around stomatal openings, but no harmful changes were noted. *L. racemosa* plays a key role in the rich diversity of mangrove ecosystems. As such, this species could, by the presence of PTEs in its leaves, be a suitable biomonitor of toxic substances in coastal environments of the world and used accordingly in strategies designed for eco-sustainable technologies.

## Introduction

Mangroves are part of marine systems substantially affected by domestic and industrial pollutants. These pollutants are mainly dumped into rivers and flow into the mangroves or they are carried by seawater to the coastal shoreline. These contaminants tend to accumulate from the large amount of organic matter in sediments and/or filtered particulate matter with the help of tree roots. Such residues may also bioaccumulate through the exposure of decomposers inhabiting the soil. Consequently, potentially toxic elements (PTEs) enter at different trophic levels of the food chain, resulting in loss and damage to biodiversity and the ecosystem^1,2^.

Brazil has one of the largest areas of mangroves, stretching about 14,000 Km^2^ along its coast. Sepetiba Bay, located in Rio de Janeiro, is an aquatic saline environment surrounded by a large restinga (coastal tropical and subtropical moist broadleaf forest in eastern Brazil) and mangrove^3^. These ecosystems have been severely impacted by anthropogenic activities since the 1970s. This bay, in particular, has experienced an increase in industrialization with the construction of the Sepetiba Industrial Complex, the Itaguaí Harbor area and the Industrial District of Santa Cruz. The “sacrifice zone” includes sites of several metallurgical, petrochemical, and pyrometallurgical smelters, as well as chemical, textile, beverage, and paper industries, all damaging such coastal ecosystems as mangroves^3–5^. Metal smelting, including Zn, Cd, Al, Fe, and alloy steel, is the major economic activity located in Sepetiba Bay's basin, followed by the chemical and paper industries^4,6^. Coroa Grande and Pedra de Guaratiba are mangrove areas in Sepetiba Bay under high anthropic pressure, unlike Maramabaia mangrove, which is located in an area of restricted access and thus considered a protected ecosystem far from the industrial center. Previous studies reported improper disposal of solid wastes containing Zn and Cd, which come from an industrial site on Madeira Island, one of the main sources of PTEs flowing into Sepetiba Bay^7^. Most PTE inputs to the bay arrive from rivers, mainly as drainage from highly industrialized and urbanized areas of Sepetiba Bay^5,7^. Cyclical periods and marine tidal currents create a dynamism that alters the physicochemical condition of mangroves, bringing pollutants directly to them^7^. PTEs also reach Sepetiba Bay through the atmosphere. Atmospheric deposition of pollutants emitted outside the bay area may further contribute to the total load of PTEs^7^.

Leaves, which sequester and store carbon and then release carbon dioxide in the mangrove ecosystem, are the main contributors of litter, comprising, in turn, the food resource of insects and arboreal crabs^2^. Leaf assessment in different ecosystems allows researchers to detect pollutants that may cause changes in plant morphoanatomy, physiology and metabolism. Different studies of mangroves have shown the presence of metals, metalloids and plasticizers in leaves^1,3,7,8^. These substances are taken up by plants through their stomata, lenticels or epicuticular waxes in leaves, but roots are the first and most common organs in contact with pollutants. In the roots, pollutants are freely diffused via apoplast and/or symplast or carried across cells to xylem from which contaminants are transported throughout the plant^9^.

*Laguncularia racemosa* (L.) Gaertn. f. (a white mangrove, Combretaceae) is an arboreal species which occurs in mangrove swamps on the Atlantic coasts of the Americas and West Africa^10^. This species is a typical pioneer found in the interior of mangroves and the transition to restinga forest. It is well known that mangrove plants can absorb, accumulate, degrade/transform and volatilize PTEs through green remediation. However, no current studies have reported on the amount of PTEs in *L. racemosa* forests from Sepetiba Bay mangroves. Apart from adaptability, plants that are good options for environmentally sustainable phytoremediation must remain healthy when subjected to environmental contaminants^11^.

Therefore, this work aimed to detect PTEs by evaluating the leaves of *L. racemosa* collected from mangroves under anthropic disturbance. To gain further insight toward an accurate diagnosis of the effects of anthropogenic pollution on mangrove stands, we evaluated leaf epicuticular wax composition, as well as morphological and anatomical traits. To accomplish this, the quantity of PTEs was determined in leaves of *L. racemosa* growing in mangrove sites—Coroa Grande (CG), Pedra de Guaratiba (PG) and Marambaia (M, control area) around Sepetiba Bay, Rio de Janeiro. The final results of this assessment will determine if *L. racemosa* has potential as a bioindicator for mangroves polluted by PTEs and, as such, play a role in green remediation and protection of these ecosystems.

## Methods

### Study sites

**Figure 1:**
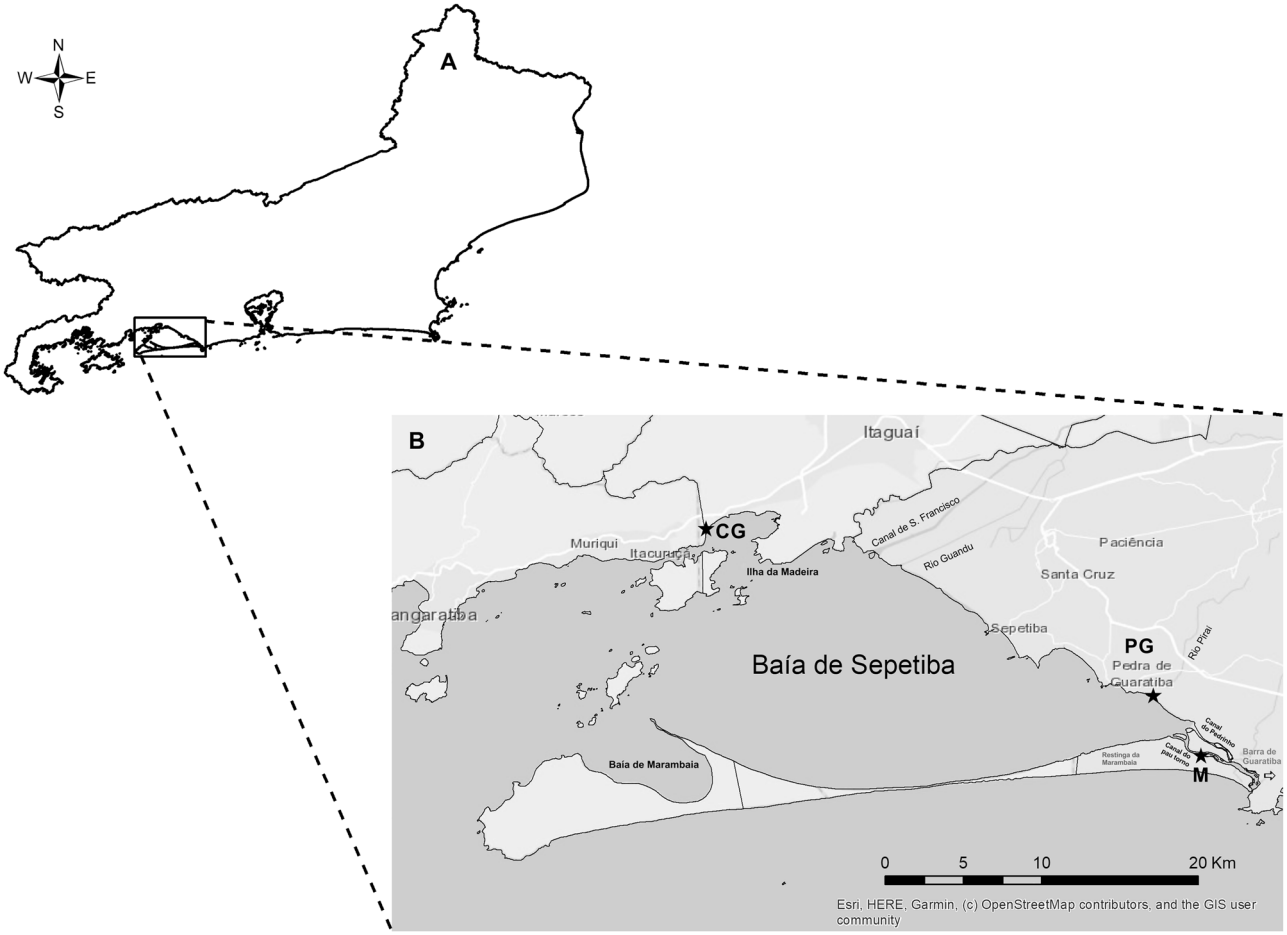
(**A**) Rio de Janeiro City. (**B**) Mangroves around Sepetiba Bay where *Laguncularia racemosa* leaves were collected (*). Black stars indicate areas of plant collection in mangroves: Coroa Grande—CG (Itaguaí City), Pedra de Guaratiba—PG (Rio de Janeiro City) and Marambaia—M (Rio de Janeiro City). Industrial centers are found in Itaguaí and Santa Cruz. ArcMap 10.6 program.

Mangrove sites selected for this study are located around Sepetiba Bay (Rio de Janeiro, Brazil). The Bay area is 305 km^2^. Three mangrove sites (Fig. 1) were chosen based on ease of accessibility to different degrees of exposure to pollution. Coroa Grande-CG (22°54′42.23"S and 43°52′48.88" W) and Pedra de Guaratiba-PG (23° 0′27.98"S and 43°37′22.41"W) have a high incidence of industrial activity (Fig. 1), mainly from the metallurgical sector, and Marambaia-M (23° 2′ 32.46ʺ S and 43° 35′ 43.99ʺ W) is considered a control area owing to its well-preserved ecosystem. Sepetiba Bay has brackish water according to resolution n. 357/2005 of CONAMA (Brazil’s Environmental National Council) and taking into account dissolved oxygen (DO), salinity and pH (Table 1). Recent studies have already shown the content of PTEs in sediments of Sepetiba Bay^12^ (Table 1).

**Table 1:** Concentrations of potentially toxic elements in sediments and physicochemical properties of water in mangroves of Sepetiba Bay.

PTEs (symbol)	Mangrove sediments* (mg/kg)
Chrome (Cr)	11.12
Cobalt (Co)	0.25
Copper (Cu)	3.73
Molybdenum (Mo)	80.03
Manganese (Mn)	156.25
Zinc (Zn)	278.36
Physical parameters**	
pH	6.5–8.1
Salinity (%)	20–33
Conductivity (mS/cm)	17.7–21.9
DO (mL/L)	7.0–8.3
Turbidity (UNT)	9.1–11.7

*Data analyzed by ICP-OES refer to samples (n = 5) of up to 20 cm depth of sediment from Sepetiba Bay mangrove according to Flores et al.^12^. ** Silva^13^.

### Sampling procedures

At each study site, five randomly selected *Laguncularia racemosa* (L.) Gaertn. f. trees during the reproductive stage were chosen as representatives of five monitored plots. Leaves from each individual were taken from three different mangrove sites between June and July (average of 16–26 °C temperature and 48–62 mm pluviosity). *Laguncularia racemosa* was previously identified, and a voucher specimen is deposited at the Herbarium RBR at UFRRJ, Rio de Janeiro, Brazil.

Ten leaves of each individual were used for each mangrove area to determine leaf area (LA) and leaf perimeter, using ImageJ, v. 1.42q. For dry weight, leaves were placed in a 50 °C oven until constant weight. Specific leaf area (SLA) was calculated using the ratio of LA to the corresponding leaf dry weight.

### Acid digestion and analysis of minerals by spectrometry

Leaf samples were dried in an oven at 60 °C with air circulation and then submitted to digestion with nitric acid 65% ultrapure (Merck). To an Erlenmeyer flask were transferred 0.2 g of dried leaves to which 10 mL of HNO_3_ were added under an exhaust hood. Samples were placed in an ultrasound apparatus for 30 min. Following this, samples were transferred to an oven to dry at 60 °C for 60 min. After cooling, an additional 5 mL of HNO_3_ were added and evaporated off at 95 ± 5 °C for 2 more hours. Digested samples were filtered through a quantitative filter (n° 40), diluted to 50 mL with distilled-deionized water plus 5 drops of HNO_3_, and stored in Falcon tubes at low temperature until analysis. Samples were analyzed by Inductively Coupled Plasma-Optical Emission Spectrometry (ICP-OES) 2100 using a PerkinElmer Optima 7300 V ICP/OES apparatus. To prepare the standard curves, a multi-element standard concentration of 100 mg/L with the same elements was used. Calibration curves were adjusted according to the analysis of elements. Data were obtained by means of three replicates from three to five individuals of each mangrove area.

### Extraction and chemical analysis of epicuticular waxes

Leaves were placed in a separate preweighed flask containing 50 mL of chloroform (spectroscopy grade; Tedia) and maintained for 30 s under gentle manual agitation. This procedure extracts only surface chloroform-soluble compounds without disturbing the leaf interior. Chloroform extracts were maintained at room temperature for solvent evaporation to obtain the solid residue. The amount of wax was expressed per unit LA (µg/mm^2^). The experiment was conducted in a randomized design with ten replicates for each individual and locality. Two mg of dried extract were dissolved in 200 mL of chloroform. Subsequently, 1 µL was injected into a gas chromatograph (GC-2010-Shimadzu) equipped with a flame ionization detector (FID). Analyses were performed on a DB-1MS capillary column (30 m × 0.25 mm × 0.2 mm), using He as carrier gas at 1 mL/min and a split ratio (50:1). Temperature was increased by 10 °C/min from 140 to 300 °C and then maintained at 300 °C for 15 min. The injector was maintained at 290 °C and the detector at 300 °C. Quantification was performed from GC/FID profiles, using relative area (%). Identification of *n*-alkanes was based on injection of commercial standards (alkane standard solution C21-C40-Sigma Fluka). Analysis of a subsample was performed in a GC/MS QP 2010 Plus Shimadzu mass detector, using the same operating conditions as those noted above (except the column; ZB-5MS column 30 m × 0.25 mm × 0.2 mm), and the MS was scanned for 50–650 amu at 2 s/decade with an electron impact ionization potential of 70 eV. Triterpenoid compounds were identified by comparing the corresponding mass spectra with library data (Spectrum Libraries: NIST05.LIB) complemented with proton nuclear magnetic resonance (1H NMR) spectrometry (Bruker DRX 400 MHz), using deuterated chloroform as solvent. Only signals with relative abundance above 5% were considered.

### Microscopy analysis

For light microscopy observations and measurements, three leaves from each site were fixed in FAA (formaldehyde, ethanol, acetic acid, 10%:50%:5%), dehydrated in ethanol series, and stored in 70% ethanol. Middle portions of leaves were embedded in glycolmethacrylate (Historesin), cross-sectioned (8 µm thickness) in a rotary microtome (Leica RM 2155), and stained with Toluidine Blue 1%. Photomicrographs of leaf sections were made using light microscopy (Leica) fitted with a digital camera. Measurements were taken from images obtained from leaf cross sections using a microscope (Nikon Eclipse CI) equipped with a digital camera (Moticam Pro 252b). To identify the presence of zinc in plant tissues, free-hand cuts were performed on collected leaves, dehydrated at room temperature and exposed to Zincon (Sigma) reagent^4^. For scanning electron microscopy observations (SEM), segments of dry leaves were analyzed with a JEOL-JSM 6390 LV scanning electron microscopy (JEOL, Tokyo, Japan). To analyze the epidermis in frontal view and count the secretory glands/mm^2^, we prepared leaf peels mounted in 50% glycerin on semi-permanent slides.

### Statistical analyses

Principal Component Analysis (PCA) was performed in a bidimensional plane (component/dimension 1 and 2) in order to identify the main chemical elements responsible for data variance in leaves collected throughout the study site. To perform PCA, both "FactoMineR" and "factoextra" R packages were used^14^. Hence, we applied the Kruskal–Wallis test for quantitative comparisons of the main chemical elements detected in the leaves of *L. racemosa* among the three mangrove sites. When a significant difference was detected (*P* < 0.05), we applied pairwise comparisons using the Wilcoxon rank sum test with continuity correction^15^.

We applied one-way ANOVA to compare fresh and dry weight (g) and LA (mm^2^) of *L. racemosa* among the three mangrove sites. When a significant difference was detected (*P* < 0.05), we performed pairwise comparisons using Tukey's post hoc test. To perform these analyses, we used the “multcomp” R package^16^. We also used one-way ANOVA with Tukey's post hoc test to compare difference in measurements between the intercostal region (epidermis and mesophyll traits) and main rib (epidermis and vascular system traits) of *L. racemosa* leaves among the three sampled mangroves. Statistical analyses were performed in R, version 4.0.2^17^.

### Plant material

All methods were performed in accordance with relevant guidelines and regulations.

## Results

### Morphometric leaf traits

**Figure 2:**
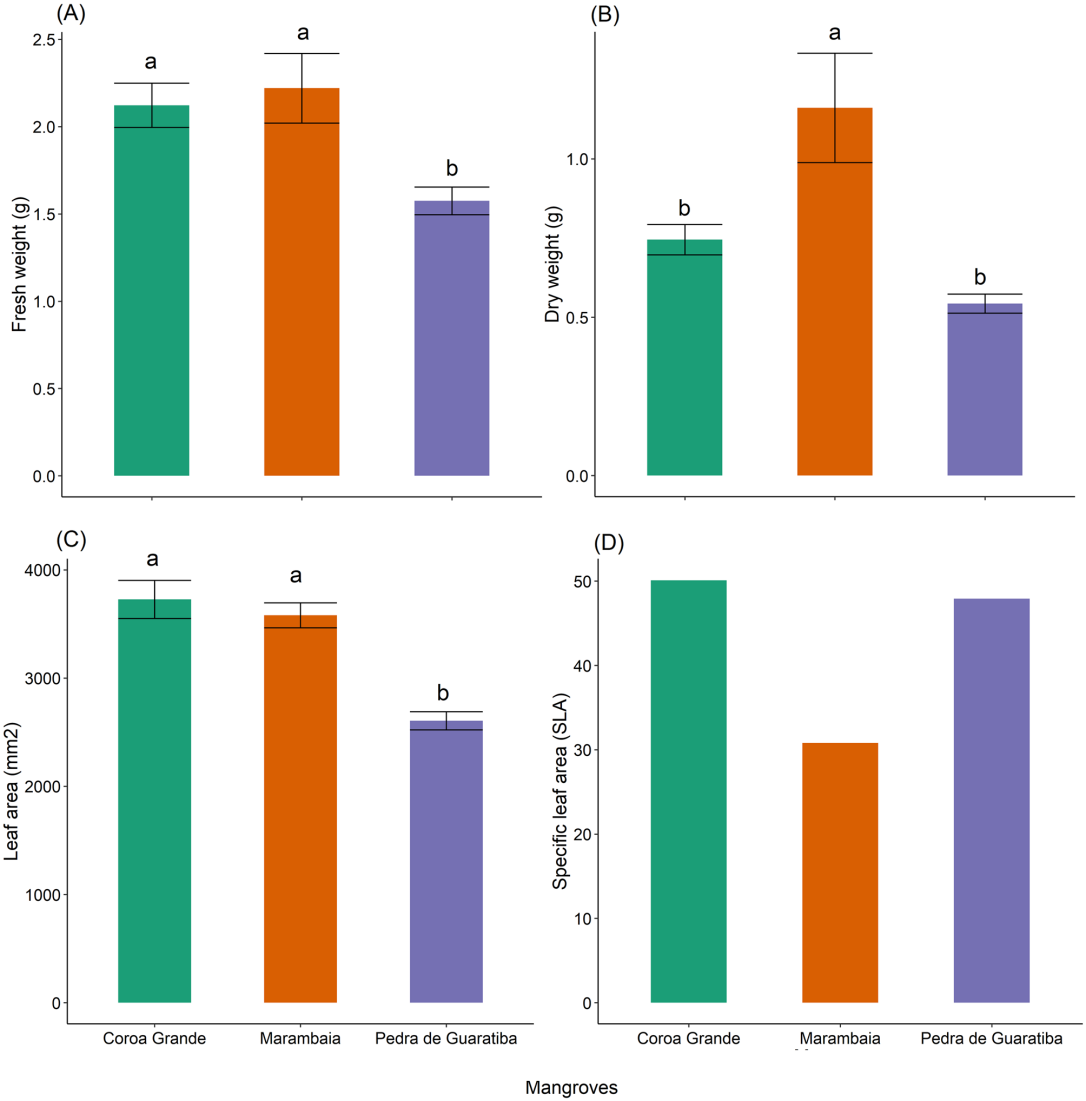
(**A**) Fresh and (**B**) dry weight (g), (**C**) leaf area (LA, mm^2^) and (**D**) specific leaf area (SLA) of *Laguncularia racemosa* collected in mangroves of Marambaia (M), Coroa Grande (CG) and Pedra de Guaratiba (PG). Different letters indicate significant difference by Tukey’s HSD test (*P*_*adj*_ < 0.05).

Sample plasticity was observed in the biometric leaf traits of *L. racemosa* among Marambaia (M), Coroa Grande (CG), and Pedra de Guaratiba (PG) (Fig. 2). Fresh leaf weight ranged from 1.16 g in CG to 3.33 g in M, while dry leaf weight ranged from 0.42 g in CG to 2.12 g in M. Fresh leaf weight of *L. racemosa* from M (2.22 ± 0.63 g) and CG (2.12 ± 0.56 g) was found to be statistically similar (P_adj_ = 0.87), but also significantly higher than that from PG (1.57 ± 0.25 g) (P_adj_ < 0.05) (Fig. 2a). Dry leaf weight of *L. racemosa* from CG (0.74 ± 0.21 g) and PG (0.54 ± 0.09 g) was found to be statistically similar (P_adj_ = 0.23), but lighter than that from M (1.16 ± 0.54 g) (P_adj_ < 0.05) (Fig. 2b). LA ranged from 2192.90 mm^2^ in PG to 4481.00 mm^2^ in CG. LA from samples of *L. racemosa* collected in M (3580.00 ± 0.364 mm^2^) and CG (3627.00 ± 0.613 mm^2^) were statistically similar (P_adj_ = 0.73), but significantly higher (P_adj_ = 0.001) than that from PG (2605.00 ± 0.262 mm^2^) (Fig. 2c). Leaf perimeter was 271.0 mm (CG), 253.2 mm (PG) and 282.5 mm (M). However, leaves from M presented lower specific leaf area (SLA = 30.8) than that from either CG (SLA = 50.1) or PG (SLA = 47.9) (Fig. 2d).

### Mineral content/elemental composition of leaves

The two main components identified by PCA explain, respectively, 27.4% and 21.3% of the variance in the number of chemical elements in *L. racemosa* leaves collected in the three areas. At a 95% confidence level, the samples do not form distinct clusters for each mangrove area in a two-dimensional plane. The amount (mg/kg) of main elements responsible for variance in component 1, consisting of Ba, Na, Ca, Mn, Ni, Pb, Cd, as well as component 2, consisting of B, Cd, Ni, Pb, Ca, Zn and Cu, did not differ significantly among leaf samples collected in CG, M and PG (P_adj_ > 0.05; see Supplementary Fig. S1online; Table 2).

**Table 2:** ICP-OES analysis of mineral content in leaves (mg/kg) of *Laguncularia racemosa* collected in Coroa Grande (CG), Pedra de Guaratiba (PG) and Marambaia (M) mangroves, Rio de Janeiro, Brazil.

Minerals (mg/kg)	CG	PG	M	Reference plants^†^
Metals				
Al*	0.74 ± 0.45	0.38 ± 0.1	0.37 ± 0.061	80
As*	0.002 ± 0	0.002 ± 0	0.002 ± 0	
Ba	0.027 ± 0.004 a	0.039 ± 0.0005 a	0.025 ± 0.004 a	40
Be	0.002 ± 0	0.002 ± 0	0.002 ± 0	
Cd*	0.032 ± 0.01 a	0.016 ± 0.01 a	0.009 ± 0.02 a	0.03–0.9
Pb*	0.32 ± 0.13 a	0.16 ± 0.12 a	0.10 ± 0.17 a	1.54
Li	0.005 ± 0	0.005 ± 0	0.005 ± 0	
Sr	0.29 ± 0.036	0.45 ± 0.147	0.24 ± 0.07	
Cr*	0.005 ± 0	0.005 ± 0	0.005 ± 0	
Sn	0.01 ± 0	0.01 ± 0	0.01 ± 0	
Ti	0.006 ± 0.003	0.005 ± 0	0.005 ± 0	5.0
V*	0.005 ± 0	0.005 ± 0	0.005 ± 0	
Micronutrients				
Co	0.005 ± 0	0.005 ± 0	0.005 ± 0	0.2
B	0.10 ± 0 a	0.21 ± 0.06 a	0.35 ± 0.79 a	
Mn*	0.091 ± 0.007 a	0.20 ± 0.02 a	0.13 ± 0.033 a	15–100
Mo*	0.01 ± 0	0.1 ± 1.69	0.01 ± 0	
Ni*	0.005 ± 0.0a	0.087 ± 0.141a	0.005 ± 0.004a	3.10
Zn*	0.26 ± 0.11a	0.33 ± 0.025a	0.25 ± 0.16a	50.80
Cu*	0.028 ± 0.005a	0026 ± 0.002a	0.018 ± 0.003a	10.0
Fe*	0.33 ± 0.08	0.51 ± 0.006	0.36 ± 0.09	89.22
Macronutrients				
Na	68.71 ± 5.512a	38.33 ± 4.73a	92.0 ± 11.56a	
Mg	12.718 ± 1.13	14.034 ± 3.83	9.71 ± 3.006	
Ca	37.25 ± 3.10a	69.23 ± 19.85a	42.18 ± 8.50a	
K	29.126 ± 1.78	26.025 ± 2.63	29.063 ± 7.23	
Se*	0.007 ± 0.001	0.006 ± 0.001	0.006 ± 0.001	
Si	0.5 ± 0.07	0.55 ± 0.04	0.76 ± 0.24	0.1
Sb*	0.002 ± 0.001	0.002 ± 0	0.002 ± 0	(< 0.5) 1.5^††^

*PTEs. ^†^Markert^18^, values in mg/Kg dry weight. ^††^Levels in leaves of different plants: Pérez-Sirvent et al.^19^ Equal lowercase letters in the same row indicate no significant difference (*P*_adj_ > 0.05). Unaccompanied elements of these letters were not statistically compared among sites.

Table 2 shows the mean concentrations of minerals (mg/L) in *L. racemosa* leaves. The content of Cd, Zn, As, Al, Cr, Cu, Pb and Se was similar in leaf samples from all three sites. Leaf samples from PG and CG showed similar results in terms of the order of bioaccumulation: Fe > Al > Zn > Mn > Pb > Cu > Cd, with Cd varying by having higher content in CG compared to M having the lowest content. The order of accumulation of PTEs in leaves of *L. racemosa* from M was Si > Al > Fe > Zn > Mn. Al and Cd had higher concentration in CG, but without statistical differences from PG and M. For comparison, the total concentration of PTEs within a normal range for plants is presented in Table 2, which specifically shows the normal values for plants as 0.03–0.9 mg/kg for Cd and 50.8 mg/kg for Zn. When compared to chemical fingerprinting of reference plants, *L. racemosa* leaves contain a lower concentration of PTEs.

### Chemical composition of epicuticular wax

The amount of triterpene content in epicuticular wax per leaf follows the descending order of Coroa Grande (CG) > Pedra de Guaratiba (PG) mangrove (Table 3). Epicuticular wax composition presented the major pentacyclic triterpenes lupeol (fagarasterol) and β-amyrin in leaves from CG and PG mangroves. These compounds gave evidence that the chemical profiles of *L. racemosa* leaf waxes were consistent. The n-alkanes hentriacontane and 8-octadecanone were identified in leaves from CG, while tetratetracontane was only found in leaf samples from PG. None of the analyzed wax components was detected in samples from Marambaia (M).

**Table 3:** Composition of triterpenes identified in epicuticular wax of *Laguncularia racemosa* leaves.

Constituent	RT (min)	Relative area (%)*		
		CG	PG	M
Hentriacontane	61.50	28.01	nd	nd
Tetratetracontane	66.17	nd	10.93	nd
8-octadecanone	70.16	6.31	nd	nd
*β*-amyrin	74.63	8.81	16.35	nd
Lupeol (fagarasterol)	75.57	53.63	41.61	nd
Total		62.44	57.96	nd

*Mean amount of wax extracted with chloroform. RT—Retention time obtained by GC. *nd* Not detected. *CG* Coroa Grande; *PG* Pedra de Guaratiba, and *M* Marambaia.

### Anatomical traits of leaves

**Figure 3:**
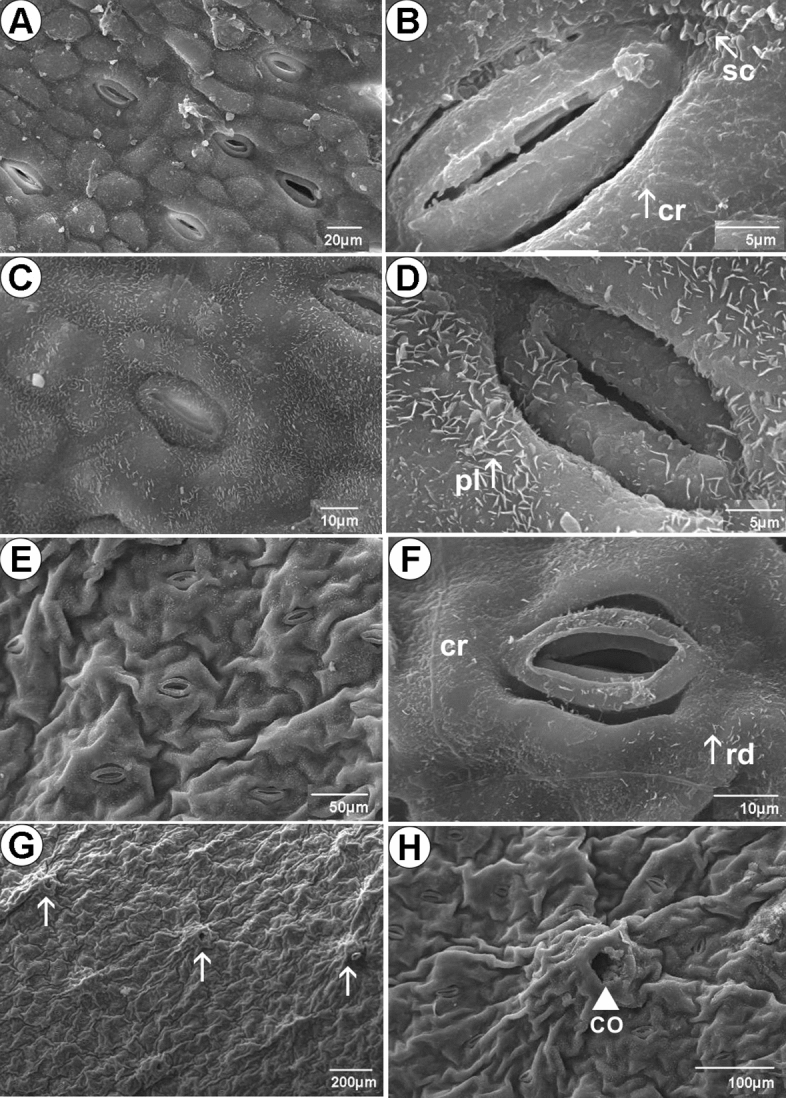
Leaf micromorphology of *Laguncularia racemosa* from three mangrove sites in Rio de Janeiro State (Brazil), showing different epicuticular wax deposition, stomata and pore of salt secretory structure located in a cavity by electronic microscopy. (**A**,**B**) Marambaia (M) mangrove: epicuticular wax layer in crusts (cr) and scales near a stomatal pore. (**C**,**D**) Pedra de Guaratiba (PG) mangrove: epicuticular wax in plates (pl) in all surfaces, including next to stomata. (**E**–**H**) Coroa Grande (CG) mangrove: epicuticular wax in crusts with some rodlets (rd) over ordinary epidermal cells and stoma. (**G**,**H**): abaxial epidermis from CG mangrove showing opening of epidermal cavity (co) where secretory multicellular glandular trichomes are located. (**A**,**C**–**H**) Abaxial surface. (**B**) Adaxial surface.

**Figure 4:**
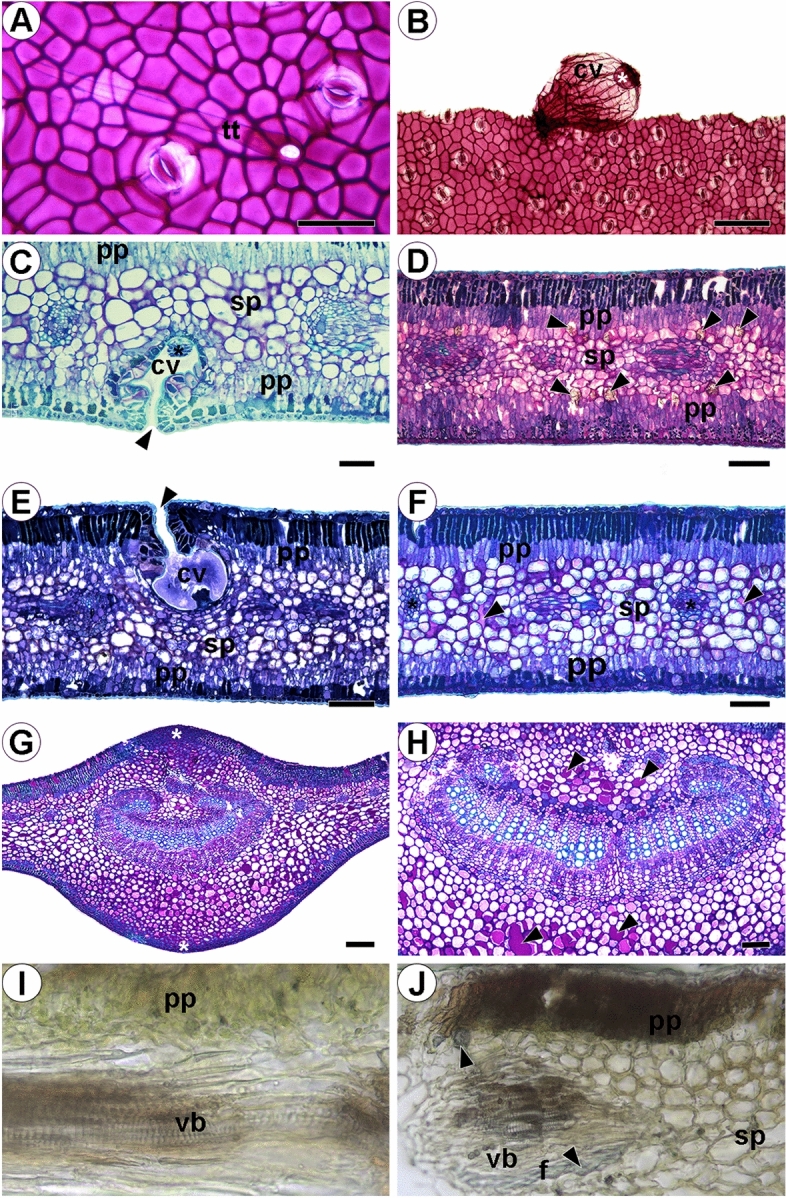
Light microscopy anatomical analysis of *Laguncularia racemosa* leaves collected in different mangroves in Rio de Janeiro (Brazil). (**A**,**B**) Frontal view of epidermis showing ordinary epidermal cells, stomata, unicellular non-glandular trichome (tt), and secretory cavity (cv) after removal of mesophyll with a salt secretory trichome at the bottom (*). (**C**) Cross section showing cavity (cv) on the abaxial epidermal surface where the salt secretory trichome (*) is located, with opening to external environment (black arrowhead), palisade parenchyma (pp) and spongy parenchyma (sp). (**D**) Leaf from Coroa Grande (CG): (pp) and (sp), crystal idioblasts with druses (black arrowheads). (**E**) Leaf from Pedra de Guaratiba (PG): adaxial secretory cavity (cv) with pore (black arrowheads). (**F**) Leaf from Marambaia (M): (pp), (sp), vascular bundles (vb); black arrowheads point to mucilage in spongy parenchyma. (**G**) Midvein cross section showing cortical and medullary parenchyma storing phenolic compounds (*). (**H**) Main vascular bundle evidencing phenolic idioblasts and mucilage (black arrowheads). (**I**) Cross section taken from a plant growing in M and exposed to Zincon. (**J**) Cross section taken from a plant growing in CG and exposed to Zincon, evidencing Zn in fibers and idioblasts with druse crystals. (**A**,**E**) CG mangrove; (**B**,**F**,**H**) M mangrove and (**D**,**G**) PG mangrove. **A**,**B** = 500 μm, **C**, **G**= 20 μm, **D**, **E**, **F**, **H**, **J**= 100 μm, **I**= 50 μm.

**Figure 5:**
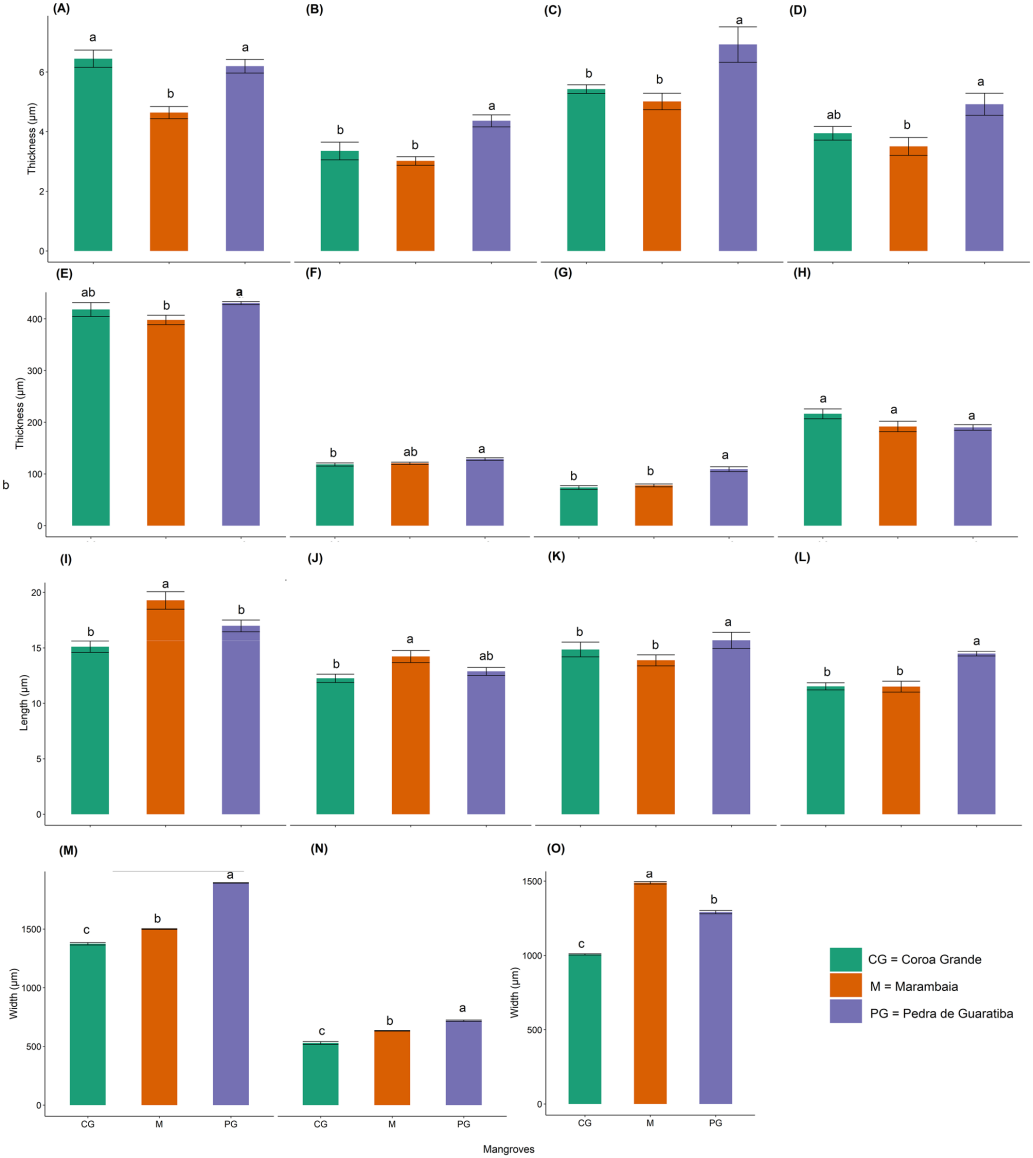
Light microscopy anatomical analysis of *Laguncularia racemosa* leaves collected in different mangroves in Rio de Janeiro (Brazil). Intercostal region: (**A**) thickness of adaxial wax + cuticle layer; (**B**) thickness of abaxial wax + cuticle layer; (**E**) thickness of mesophyll; (**F**) thickness of adaxial palisade parenchyma; (**G**) thickness of abaxial palisade parenchyma; (**H**) thickness of water storage parenchyma; (**I**) length of adaxial epidermal cells; (**J**) length of abaxial epidermal cells. Midvein region: (**C**) thickness of adaxial wax + cuticle; (**D**) thickness of abaxial wax + cuticle; (**K**) length of adaxial epidermal cells; (**L**) length of abaxial epidermal cells; (**M**) length of midvein region; (**N**) length of midvein; (**O**) width of midvein. Different letters indicate significant difference by Tukey’s HSD test (*P*_*adj*_ < 0.05).

The epicuticular wax layer covering the leaf surface presented distinct patterns, i.e., plates, granules, rodlets and crusts, according to the site of collection (Fig. 3). Leaves from Marambaia (M) presented epicuticular wax in crusts and granules covering most of the surface area, but in scales near stomata (Fig. 3a,b). In leaves from Pedra de Guaratiba (PG), the wax layer consisted of crusts and plates throughout the surface, including stomatal cells (Fig. 3c,d). In leaves from Coroa Grande (CG), the epicuticular wax consisted of granules over common epidermal cells, but a rod pattern near stomata (Fig. 3e,f). Pores of the cavity where salt secretory glands are located were seen on both surfaces (Figs. 3g,h and 4). In cross section, the epicuticular wax and cuticular layer are not easily distinguished under light microscopy (Fig. 4), so they were measured together. The thickness of wax and cuticular layer together on adaxial surface was smaller in M (4.64 ± 0.78 µm, Fig. 5a) compared to that in PG (6.19 ± 0.88 µm) and CG (6.45 ± 1.12 µm) (*P*_*adj*_ < 0.001) (Fig. 5b). On the abaxial surface, plants from CG (3.35 ± 1.15 µm) and M (3.02 ± 0.55 µm) presented less thick wax and cuticular layer together than that found in plants from PG (4.36 ± 0.77 µm) (*P*_*adj*_ < 0.05) (Fig. 5b).

*Laguncularia racemosa* has amphistomatic leaves; the ordinary epidermal cells present straight walls (Fig. 4a,b). Unicellular trichomes were detected only in plants collected in CG (Fig. 4a). In cross section, the epidermis consisted of a single layer of epidermal cells (Fig. 4c–f). Common epidermal cells store phenolic compounds more intensely, as detected in plants from CG (Fig. 5e). The adaxial epidermal cells in plants from M were longer (19.30 ± 3.07 µm) than those from PG (17.00 ± 2.03 µm) and CG (15.10 ± 2.00 µm) *(P*_*adj*_ < 0.05; Fig. 5I). The length of abaxial epidermal cells from plants of the three mangroves were, however, found to be statistically similar (*P*_*ad*_ > 0.05), even though longer abaxial epidermal cells were found in M (14.20 ± 2.09 µm) compared to abaxial epidermal cells from CG (12.2 ± 1.46 µm) (*P*_*adj*_ = 0.007; Fig. 5j). Salt-secreting glands are located in epidermal cavities that invade the mesophyll (Fig. 4b,c–e). The number of glandular trichomes was consistent between the two faces of the leaf and also among the three sites evaluated (2–3 glands/mm^2^). The salt-secreting gland is supported by short peduncle cells with 3–4 (basal cells) sustaining the secretory cells that form a nearly globose structure (Fig. 4b,c,e). The epidermal cells that surround the opening of the cavity (pore) are palisade-like in cross section, with tabular shape, containing phenolics (Fig. 4c,e). The salts, mucilage and other substances excreted by this secretory structure first accumulate in the cavity and thereafter are most likely released to the external environment through the epidermal pore (Figs. 3g,h and 4e).

Found to be heterogeneous and isolateral in leaves from the three sites investigated, the mesophyll was comprised of palisade parenchyma (adaxial and abaxial) and 4–5 layers of spongy water storage parenchyma (Fig. 4d–f). The thickness of the mesophyll was greater in leaves from PG (430.67 ± 11.15 µm) compared to plants from M (397.94 ± 35.33 µm) (*P*_*adj*_ = 0.049), but mesophyll thickness in both PG and M was similar to that found in CG (418.13 ± 51.86 µm) (*P*_*adj*_ > 0.05; Fig. 5e). Longer adaxial palisade parenchyma cells were observed in PG (128.77 ± 9.84 µm) compared to those in CG (118.43 ± 11.60 µm) (*P*_*adj*_ = 0.019), but adaxial palisade parenchyma cells in both PG and CG were similar to those found in M (120.68 ± 8.40 µm) (*P*_*adj*_ > 0.05; Fig. 5f). In the first layer of palisade parenchyma under both sides of the epidermis, a strong reaction to phenolic compounds was observed (Fig. 5d–f). Abaxial palisade parenchyma cells were longer in PG leaves (109.18 ± 17.79 µm) than those observed in M (77.84 ± 10.70 µm) and CG (73.73 ± 14.00 µm) (Fig. 5g) (*P*_*adj*_ < 0.0001; see Supplementary Fig. S1 online). Water storage parenchyma was composed of voluminous cells with few, or no, chloroplasts (Fig. 5c–f). The thickness of this tissue ranged from 78.04 µm in leaves from M to 285.15 µm in leaves from CG, but no significant difference was found among the three sites (*P*_*adj*_ > 0.05; Fig. 5h). In all samples evaluated, the cell wall of water storage parenchyma presented an intense reaction, indicating the presence of pectic mucilage. Mucilage was spread throughout cells in the mesophyll (Fig. 4f). Idioblasts with calcium oxalate crystals (druses) were observed in cells of spongy parenchyma and were found to be visually more abundant in PG leaves. Calcium oxalate crystals (druses) presented positive reaction to the Zincon histochemical test with more intensity in leaves from CG and PG, indicating the presence of this PTE (Fig. 4i,j). Vascular bundles were observed to be the collateral type with fibers in the most developed units forming a cap protecting the phloem (Fig. 4c). Positive reaction to the presence of zinc was observed in vascular fibers in CG and PG leaves (Fig. 4l,j). A parenchymal sheath (endodermis) was present and was best observed in the larger bundles. It did not touch the epidermis (Fig. 4c,d). Phenolic compounds and idioblasts with druse crystals of calcium oxalate are typically associated with vascular tissue. However, we did not observe any qualitative differences in the vascular system of leaf samples from the three collection sites.

The cross section of the main vein shows epidermal cells on the adaxial face with a rectangular shape (Fig. 5g), while those on the abaxial face were square to papillose. The thickness of the adaxial cuticle layer and length of the adaxial epidermal cells were greater and longer, respectively, in PG when compared to the same leaf traits measured in CG and M (*P*_*adj*_ < 0.05; Fig. 5c,k). The abaxial epidermal cells were also longer from PG (14.5 ± 0.47 µm) than those from CG (11.5 ± 0.73 µm) and M leaves (11.5 ± 1.10 µm) (*P*_*adj*_ < 0.05; Fig. 5l). However, in the abaxial layer, cuticle thickness just differed between PG and M leaves (*P*_*adj*_ = 0.016; Fig. 5d). Under adaxial and abaxial epidermal sides, we observed 6–8 layers and 1–2 layers, respectively, of regular parenchyma storing phenolic compounds and crystals. In the region of the main vein, the parenchyma cells were observed to be isodiametric and separated by intercellular spaces filled with mucilage (Fig. 5g). The main vascular bundle was the collateral type, with a reniform shape, surrounded by a few fibers and collenchyma (Figs. 4g–h and 5h). Both main rib and vascular bundle were longer in PG than those traits in M and CG plants (*P*_*adj*_ < 0.00001; Fig. 5m,n). However, the main vascular bundle was larger in M (1489.04 ± 19.51 µm) compared to that in PG (1291.36 ± 24.17 µm) and CG (1006.91 ± 8.58 µm) (P_adj_ < 0.00001; Fig. 5o). Tissue composition and organization indicate that this region of the leaf blade plays an important role in leaf support through water, phenolic and mineral (calcium oxalate crystals—druses) reserves.

## Discussion

Sepetiba Bay is a coastal area sensitive to regional environmental changes that can create an ecotone where interactions between land and water take place. One of the many factors altering the concentration of minerals and PTEs in Sepetiba Bay is the frequent movement of tidewater and sediment from water entering the bay. Hence, PTEs, with added mobility, may also change in the short term. In addition, industrial activities have production rates that can alternately reduce and increase pollution^20,21^.

The high concentration of Na > Cl > K > Ca > K in leaves of *L. racemosa* collected in Marambaia (M), Coroa Grande (CG) and Pedra de Guaratiba (PG) is common in saline ecosystems that have input of seawater. Nonetheless, the amount of Na is three times greater in leaves of M compared to leaves examined from CG and PG mangroves. This result could be attributed to the degree of salinity in the region where high hydrodynamics is known to significantly change salinity. Soil salinity can interfere with the uptake and exchange of PTEs in the soil–plant system, which, in turn, is reflected in the morphology and structure of plants^22^. Moreover, PTEs constitute one of the main abiotic agents related to growth reduction and alteration in physiological processes^23^. In this sense, we found that the average dry weight showed the following descending order: M > CG > PG (Fig. 2). Also, SLA was lower in leaves from M than SLA from either CG or PG mangroves, suggesting the influence of pollutants on plant development since CG and PG are virtually surrounded by widespread industrialization. Even so, the limits of PTEs considered safe in plants were not exceeded. Still, SLA can be an important indicator of the impact of pollution in the environment and, thus, can play an important role in the protection and adaptation of plants^24^. Leaf traits are very significant in studies reporting on environmental quality because such traits are involved in the uptake, accumulation and/or metabolization of different contaminants^25^. Such bioaccumulation of pollutants, when combined with the canopy density of trees like *L. racemosa*, contribute to improving the green strategies of mangroves around the world.

PTE contents in leaves of mangroves sampled in this study are lower than those observed in normal reference plants grown in uncontaminated soils. Machado et al.^26^ conducted a study with *L. racemosa* in Guanabara Bay (RJ) and found that metals tended to accumulate in chemical forms that reduced their mobility and, hence, absorption by biota. Moreover, a low translocation of PTEs was observed from the leaves of litter to other trophic levels, suggesting their low bioavailability. When leaves fall in the mangrove substrate, a low amount of PTEs present in the leaf litter is returned to the soil by decomposition and mineralization processes, becoming bioavailable in the environment in a way that culminates in biomagnification processes along the food chain. Still, the release of PTEs from fallen leaves is low^27^.

As the mangrove area nearest the industrial complex, it follows that CG would experience high values of Zn in leaves compared to the value of Zn in leaves in M, even though no differences were observed among sites. Zn occurs naturally, but it is often associated with anthropogenic activities, and it can be considered a key indicator of polluted areas, such as contaminated urban areas^28^. Also, Zn is probably associated with waste from the metallurgical plants in CG. We herein validated an association between industrial sectors engaged in metallurgy, electronics, and other industries that deposit toxic materials into the water and soil and pollution of the Sepetiba Bay system by Pb and Cd. The higher concentration of Zn (< 50 ppm) was observed in leaves of *L. racemosa* in experiments carried out by Bernini et al.^29^ from leaves collected in an estuarine mangrove of the São Mateus River, Espírito Santo, Brazil. Another study shows that *L. racemosa* accumulates high concentrations of Cr in roots, but that low mobility of this element results in correspondingly low content in leaves^30^. PTEs like Cd, Cr, Pb and Zn present high toxicity to the environment when in high quantity, and their significant bioaccumulation causes many problems in ecosystems because they are not biologically degraded, but rather accumulate in biota and in abiotic environments as trapped particulates in mangrove sediments^31^.

Other PTEs were found in leaves of *L. racemosa*, such as Ba, Si, Sb, Ti and V. These elements are employed in different industries, particularly those in the Sepetiba Bay area, and thus important to socioeconomic progress. For example, Ti, Sb and V are used in the production of metal alloys. These chemical elements are indicators of industrial development and were detected in leaf samples of *L. racemosa* for the first time. Because of the lower values from some minerals, it is possible that they do not originate from industrial sources, but, instead, are the result of biogeochemical cycling. Antimony (Sb) is also employed mainly in metal alloys^32^. The maximum value reported in leaves is 1.5 mg/kg, but in most samples, such as those examined in our data (0.2 mg/kg), the concentrations were below 0.5 mg/kg^19^ and not considered toxic.

Cuticular waxes are secreted by cells of the epidermis and deposited on all aerial plant organs, protecting them from environmental stresses^33^ and reducing excessive leaf water loss (residual transpiration)^34^. The presence of *n*-alkanes and triterpenes, as the main constituents of epicuticular wax, is recurrent among restinga and mangrove plants that use residual transpiration as a mechanism of tolerance under saline stress^3,34,35^. Pentacyclic triterpenoids, such as β-amyrin and lupeol, which are representative of the oleanane and lupane groups, are widely distributed in mangrove plants and is associated with features tolerant to salt. These triterpenes also increase the permeability that helps plants eliminate salts^36^. β-amyrin and lupeol were identified as the main components of *A. shaueriana* epicuticular wax^3^, also of *L. racemosa* leaves in this study. However, our results are different from those of Rafii et al.^37^ who verified only trace amounts of triterpenes in leaf wax of *L. racemosa* from Guyana (western Atlantic coast) and 8.5% in wax extract in a population from Gabon (eastern Atlantic coast) that did not present lupeol.

The presence of hydrocarbons in leaf epicuticular wax has proven valuable in chemotaxonomic studies as biomarkers at the species level. Alkanes were the most abundant compounds among the hydrocarbons, highlighting hentriacontane in leaf wax collected in CG. The hydrocarbons hentriacontane and octadecanone are present in leaves of *Rhizophora mangle* from Africa^37^, and hentriacontane is present in leaves of *A. shaueriana*^3^, another tree found in mangroves. The hydrocarbon hentriacontane is recorded for the epicuticular waxes of several plant species^38^, but here we show the first evidence of this hydrocarbon in the *Laguncularia* genus. As constituents of epicuticular wax, *n*-alkanes seem to be specifically related to a reduction in cuticular water loss^39^.

Mangroves are also exposed to pollution of particulate material through the atmosphere owing to their coastal distribution and proximity to urban centers. Particulate pollutants deposited on the leaf surface may be absorbed by leaf tissues, resulting in alteration of the chemical structure of epicuticular waxes, thereby causing morphoanatomical damage, such as chlorosis, necrosis, and reduction in photosynthesis and gas exchange^40^. Previous studies verified the effects of air pollution in degraded (erosion) epicuticular waxes, resulting in low drought tolerance^39^. These symptoms, which are easily visible through an analysis of the leaf surface by scanning electron microscopy (SEM), are provoked by very diverse chemical environments, such as acid rain or fog, simultaneous exposure to SO_2_ and NH_3_ or car exhaust ^41,42^. Studies of Arrivabene et al.^40^ detected a higher amount of particulate material, including iron, on the leaf surface of *A. schaueriana* and *L. racemosa*. The presence of hydrophobic epicuticular wax covering the leaf blades may be associated with absorbed chemicals, such as plastic additives, copper-based fungicides, pesticides and others^8,43^. Biotic and abiotic stresses are involved in the regulation of plant cuticle biosynthesis^30^. It has been reported that uptake of PTEs from both soil and air influences cuticular wax layer and permeability. Changes in permeability can then result in high water losses through the cuticle^44^. Evidence suggests that PTEs alter the cuticle, e.g., the expression of genes involved in Cd tolerance, while Fe deficiency was shown to reduce the amount of cuticular lipids that influence water retention, solute permeability, pathogen infection and disease resistance^30^. A positive correlation between transpiration rate and cadmium (Cd) concentration was reported in Beta vulgaris plants, potentially affecting cuticle composition. Permeability favored by the high content of triterpene, not previously seen in *L. racemosa*, contributes to the input and output of PTEs by leaf tissues and contributes to the ability of *L. racemosa* to capture PTEs not only via roots, but also through leaf surface.

Leaf anatomy of *L. racemosa* was previously analyzed regarding the description of anatomical features as adaptations to the saline ecosystem^45–47^. Here we present quantitative data about *L. racemosa* leaves from plants growing in polluted mangroves of Sepetiba Bay, as well as data about the micromorphology of epicuticular wax. *L. racemosa* has no natural barriers in roots to protect against the entry of salt that comes from seawater; instead, it is excreted through specialized glands in the leaves^48^. Evidence suggests that mature leaves of *L. racemosa* can secrete salt according to its concentration in the soil. That is, if salt increases in the substrate, then, based on the above assertion, salt secretion will be higher in leaves^49^. Through salt glands, salt-tolerant plants also can expel PTEs^40^. No leaf samples from mangroves evaluated showed morphological or structural damage, suggesting that this species is particularly tolerant to environments under high industrial activity and, therefore, exposed to a correspondingly high volume of PTEs.

Indeed, secretory glands actively eliminate salts, keeping them within certain limits^50^. In our study, the number of salt glands varied (2–3 glands/mm^2^), different from what was observed by Silva et al.^46^, who found less than 1 gland/mm^2^, and by Lima et al.^45^, who cited 2.7 (adaxial surface) and 4.2 (abaxial surface) glands/ mm^2^ to plants growing in the southern region of Brazil. Variation in the number of glands/LA may be related to the levels of salinity found in each collection site, especially from the proximity of large rivers, and, consequently, deserves to be better investigated when considering the ability to remove salts by the foliar glands. The very high content of Na in leaves from M, compared to that found in leaf samples from CG and PG mangroves, is simply suggestive of high salinity as a characteristic of the local ecosystem. This study did not verify any changes in the patterns of salt gland distribution for *L. racemosa*. The anatomical characteristics of the salt glands, which are located at the bottom of an epidermal cavity, corroborate the description provided by Francisco et al.^47^ and Dassanayake and Larkin^51^ regarding cell organization and composition.

*Laguncularia racemosa* leaves presented tissue organization similar to that described for the family and genus mentioned in the classical literature. The anatomical pattern observed in the leaf samples of *L. racemosa* differed slightly from that described by Silva et al.^46^, who evaluated the same species in a mangrove in the state of São Paulo by comparing a highly impacted area with a non-impacted one. In the epidermis, for example, the authors observed only epicuticular wax in granules. Baker^52^ suggests that epicuticular wax in the form of plates is produced by primary alcohols, such as triterpenoids, resulting in amorphous morphology, as we observed here.

In *L. racemosa* herein evaluated, we observed mesophyll similar to that found in leaves collected in northern or southern Brazil^45^, places not affected by industrial pollution, suggesting that the leaves of this species did not present significant alterations in the organization of the mesophyll, irrespective of collection site. In leaves investigated here, we observed an isolateral structure in the three sites selected, differing from the pattern cited by Lima et al.^45^ Isolateral leaves are found in plants living in sites where incident light is received from upper and lower orientations, possibly improving the photosynthetic process in an otherwise growth-limiting environment. We observed that most mesophyll was occupied by water reserve parenchyma in relation to palisade chlorophyll parenchyma, regardless of the sampled site. Water storage parenchyma is a common, but no less remarkable, tissue in halophytes and has a fundamental role in the dilution of salts absorbed together with water that could otherwise accumulate in levels toxic to the plant^50^. This result may be directly related to the saline conditions in which the species lives, suggesting that the leaves could contribute to the water support capacity. Such support would be fundamental for mangrove species subjected to constant physiological drought, similar to that proposed for parenchyma rays in *L. racemosa* wood^53^.

In the epidermis and mesophyll, we observed an intense reaction pointing to the presence of phenolic compounds in all collection areas. The occurrence of phenolic compounds, including flavonoids and derived phenolic acids, in the epidermal cells and in the palisade parenchyma close to the adaxial and abaxial surfaces may be related to photoprotection of the underlying tissues. Thus, tissue integrity would be guaranteed, even under conditions of intense luminosity, as phenolic compounds tend to relieve photo-oxidative stress and limit the formation of reactive oxygen species (ROS) in chloroplasts or reduce their formation^54^. Also, the higher phenolic content in leaf tissues from CG mangrove may be a direct result of industrial pollution in the area compared to that in M. Phenolic compounds chelate PTEs. An increase in phenolic substances, as an antioxidant response, has already been reported in plant organs from environments contaminated with PTEs^55^.

Histochemical analyses revealed the presence of Zn associated with calcium oxalate crystals (druses) in the parenchymatic tissue of the leaves. According to Silva et al.^46^, most vascular plants store some type of mineralized material, with druses being the most common form. A key function of calcium oxalate deposits in leaves, the main substance of the composition of druses, is to maintain a low concentration of Ca in the vicinity of the cells of the stoma. Ca is engaged during the opening of stomata, and the greater number of druses may be related to several changes in metabolism^46^, including those caused by PTEs stress, which explains the large number of druses in histological analyses. The presence of Zn in fibers (sclerenchyma) associated with the vascular bundle of *L. racemosa* has not been previously reported, indicating the role of this tissue in accumulating this metal.

We found that the chemical components detected in the leaves are consistent with what is expected for plants in saline environments, and these components are relatively within the levels of acceptance by competent environmental agencies and current legislation in Brazil. Here, we investigated, comparatively, morphoanatomical attributes/traits and the presence of PTEs in *L. racemosa* leaves in different mangrove areas. The absence of toxin diffusion through *L. racemosa* leaf tissue along Sepetiba Bay suggests, as noted above, that this species is resilient to the levels of pollution in that area. The adaptive response of *L. racemosa* to physicochemical features of the different collection sites resulted in significant variations between leaf morphoanatomical attributes measured. The high SLA value of *L. racemosa* collected in CG and PG mangroves is consistent with the pollution data based on proximity to the industrial complex.

The *L. racemosa* epicuticular wax studied revealed a high concentration of pentacyclic triterpenes, as well as n-alkanes, such as hentriacontane, in CG mangrove. This is the first study to report on the epicuticular wax of *L. racemosa* from mangroves of the southeastern Atlantic coast. This study showed variability in wax layer micromorphology among leaves from each mangrove investigated, including the morphological patterns mentioned for plants with the presence of terpenoids in the wax composition. The anatomical features correspond to the physiology of plants growing in saline environments under intense luminosity, as indicated by isolateral structure which, to date, has not be described for *L. racemosa*.

The detection of PTEs in *L. racemosa* leaves shows that the species has a relevant role as an ecological indicator in mangroves and that it may serve as a test organism for future studies. The degree of resilience of *L. racemosa* to PTEs, associated with its ability to absorb them from the environment, reveals the potential of the species for green remediation of contaminated mangroves. Thus, given the increasing conversion of land use and vegetative degradation on the Brazilian Atlantic coast, the suppression of *L. racemosa* would only further the loss of important ecosystem functions.

## Supplementary Information

Supplementary Information.

## Data Availability

All data generated or analyzed during this study are included in this published article.
