# Supplementary material for: Laguncularia racemosa leaves indicate the presence of potentially toxic elements in mangroves
Source: Sci Rep. 2023 Mar 24;13:4845. doi: 10.1038/s41598-023-31986-x (PMC10038979; doi:10.1038/s41598-023-31986-x)
Supplement: Supplementary file 1 — Supplementary Information. [file 41598_2023_31986_MOESM1_ESM.docx]

***Laguncularia racemosa* leaves indicate the presence of potentially toxic elements in mangroves**

Cristiane Pimentel Victório^1^, Mayara Silva dos Santos^1^, Aimêe Cordeiro Dias^1^, João Pedro Silvério Pena Bento^2^, Bruno Henrique dos Santos Ferreira^2^, Marcelo da Costa Souza^3^, Naomi Kato Simas^4^ and Rosani do Carmo de Oliveira Arruda^2^


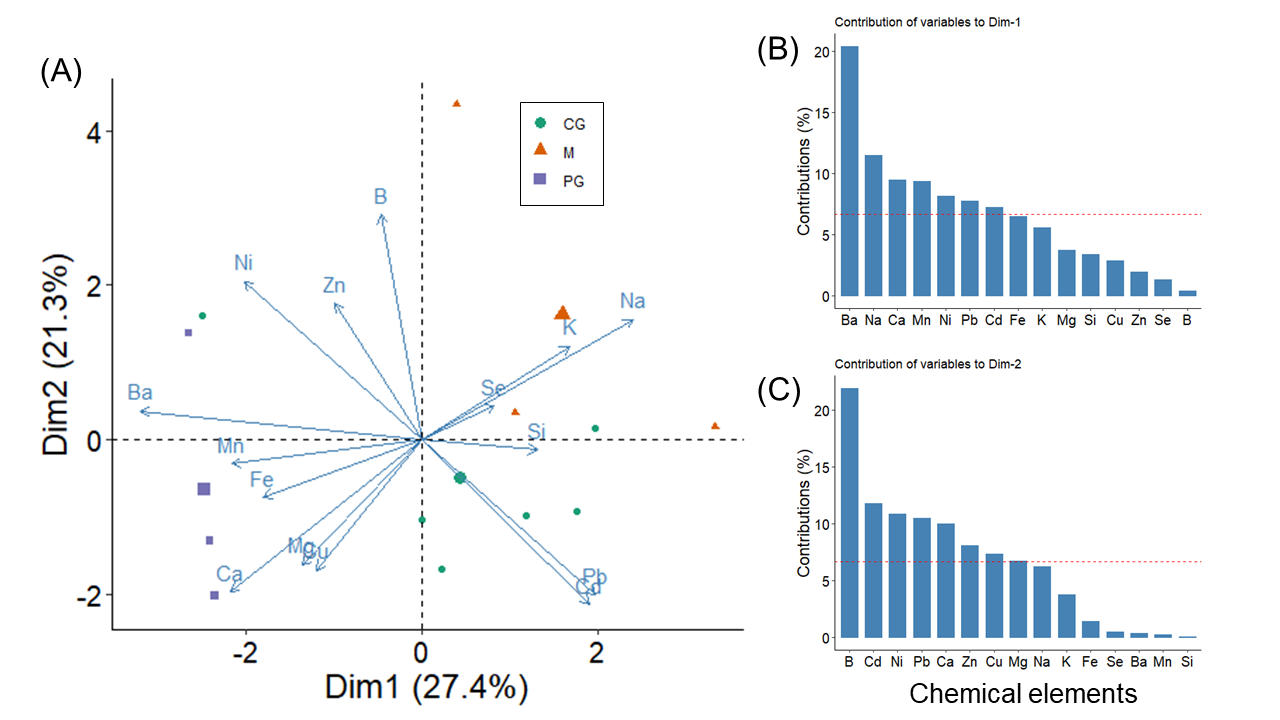


Supplementary Figure S1. Two-dimensional diagram showing the two main components explaining the variance (in percentage) in the number of chemical elements in *Laguncularia racemosa* leaves collected in three different mangrove areas; Coroa Grande (CG), Marambaia (M) and Pedra de Guaratiba (PG), in Sapetiba Bay, RJ, Brazil (A). Bars above the red line indicate the main chemical elements that contributed to the variance in dimensions 1 (B) and dimension 2 (C).
